# Assessment of kidney proximal tubular secretion in critical illness

**DOI:** 10.1172/jci.insight.145514

**Published:** 2021-05-24

**Authors:** Pavan K. Bhatraju, Xin-Ya Chai, Neha A. Sathe, John Ruzinski, Edward D. Siew, Jonathan Himmelfarb, Andrew N. Hoofnagle, Mark M. Wurfel, Bryan R. Kestenbaum

**Affiliations:** 1 Division of Pulmonary, Critical Care and Sleep Medicine and; 2 Kidney Research Institute, Division of Nephrology, Department of Medicine, University of Washington, Seattle, Washington, USA.; 3 Division of Nephrology and Hypertension, Vanderbilt University Medical Center, Vanderbilt Integrated Program for AKI, Nashville, Tennessee, USA.; 4 Tennessee Valley Health Services, Nashville VA Medical Center, Nashville, Tennessee, USA.; 5 Department of Laboratory Medicine and Pathology, University of Washington, Seattle, Washington, USA.

**Keywords:** Nephrology, Toxins/drugs/xenobiotics

## Abstract

**BACKGROUND:**

Serum creatinine concentrations (SCrs) are used to determine the presence and severity of acute kidney injury (AKI). SCr is primarily eliminated by glomerular filtration; however, most mechanisms of AKI in critical illness involve kidney proximal tubules, where tubular secretion occurs. Proximal tubular secretory clearance is not currently estimated in the intensive care unit (ICU). Our objective was to estimate the kidney clearance of secretory solutes in critically ill adults.

**METHODS:**

We collected matched blood and spot urine samples from 170 ICU patients and from a comparison group of 70 adults with normal kidney function. We measured 7 endogenously produced secretory solutes using liquid chromatography–tandem mass spectrometry. We computed a composite secretion score incorporating all 7 solutes and evaluated associations with 28-day major adverse kidney events (MAKE_28_), defined as doubling of SCr, dialysis dependence, or death.

**RESULTS:**

The urine-to-plasma ratios of 6 of 7 secretory solutes were lower in critically ill patients compared with healthy individuals after adjustment for SCr. The composite secretion score was moderately correlated with SCr and cystatin C (*r* = –0.51 and *r* = –0.53, respectively). Each SD higher composite secretion score was associated with a 25% lower risk of MAKE_28_ (95% CI 9% to 38% lower) independent of severity of illness, SCr, and tubular injury markers. Higher urine-to-plasma ratios of individual secretory solutes isovalerylglycine and tiglylglycine were associated with MAKE_28_ after accounting for multiple testing.

**CONCLUSION:**

Among critically ill adults, tubular secretory clearance is associated with adverse outcomes, and its measurement could improve assessment of kidney function and dosing of essential ICU medications.

**FUNDING:**

Grants from the National Institute of Diabetes and Digestive and Kidney Diseases (NIDDK/NIH) K23DK116967, the University of Washington Diabetes Research Center P30DK017047, an unrestricted gift to the Kidney Research Institute from the Northwest Kidney Centers, and the Vanderbilt O’Brien Kidney Center (NIDDK 5P30 DK114809-03). The funding sources had no role in the design and conduct of the study; collection, management, analysis, and interpretation of the data; and preparation, review, or approval of the manuscript.

## Graphical abstract

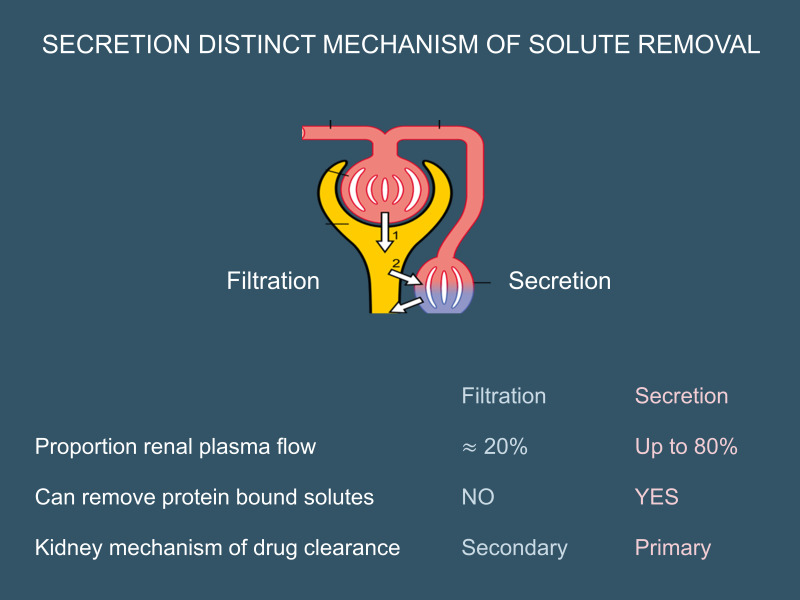

## Introduction

Acute kidney injury (AKI) is the most common form of organ dysfunction in critically ill patients and is associated with prolonged hospitalization, requirement for acute dialysis, persistent kidney dysfunction, and death (1–3). Current definitions of AKI are based on incremental changes in serum creatinine concentrations (SCrs; ref. 4). However, prognosis and recovery patterns in AKI vary substantially across individuals with similar creatinine measurements, suggesting incomplete assessment of kidney function by estimates of glomerular filtration alone (5–8).

Most underlying causes of AKI, including sepsis, ischemia-reperfusion, hypoxemia, endogenous toxins, and nephrotoxic medications, involve injury to proximal tubular epithelial cells of the kidneys (9–12). The proximal tubules reabsorb filtered substances, synthesize vital hormones, maintain saltwater and acid-base homeostasis, and secrete retained organic solutes and drugs directly into the urine. Tubular secretory clearance is essential for eliminating protein-bound substances that cannot be filtered, many of which are uremic toxins that are linked with cognitive, skeletal muscle, and cardiac impairments (13–15). Tubular secretion is also the primary kidney mechanism for eliminating hundreds of commonly administered ICU medications, including antibiotics (cephalosporins, fluoroquinolones, aminoglycosides), sedatives (propofol, benzodiazepines), and diuretics (16–18). Creating more inclusive measurements of kidney function that include tubular secretory clearance could enable more precise ICU drug dosing strategies and improve prognostic assessment.

We hypothesized that estimates of proximal tubular secretory clearance would provide additional information about kidney function that is not captured by SCr levels alone. To test this hypothesis, we used a targeted liquid chromatography–tandem mass spectrometric (LC-MS/MS) assay to quantify 7 endogenous, low molecular weight, organic solutes that are primarily cleared by tubular secretion: cinnamoylglycine, kynurenic acid, indoxyl sulfate, pyridoxic acid, xanthosine, tiglylglycine, and isovalerylglycine. We estimated tubular secretory clearance by the urine-to-plasma ratios of these solutes in matched blood and spot urine samples collected at the time of intensive care unit (ICU) admission, and we determined their association with major adverse kidney events within 28 days (MAKE_28_), which includes doubling of SCr, dialysis, and death.

## Results

### Baseline characteristics of the study cohorts.

To test the hypothesis that secretory solute urine-to-plasma (U/P) ratios are different between critically ill and healthy populations and that in critical illness secretory solute U/P ratios are associated with outcomes, matched plasma and urine samples were collected upon ICU admission from 170 participants enrolled in the Critical Illness Translational Research Cohort (CITRC) and 70 participants in the Healthy Kidney Study (HKS). Among participants in the CITRC cohort, the mean age was 50 years, 67% were men, and 79% self-identified as White race (Table 1). CITRC participants had numerous comorbidities, including diabetes (33%), hypertension (38%), and chronic kidney disease (CKD; 7%). Diagnosis of Sepsis-3 was common (85%), as were the use of mechanical ventilation (49%) and septic shock (49%). The mean SOFA score on day 1 was 7.1 ± 4.5, and the mean SCr concentration on day 1 was 1.3 ± 1.0 mg/dL. Among participants in the HKS cohort, the mean age was 50 years, 50% were male, and the mean SCr concentration was 0.82 ± 0.19 mg/dL.

### Comparison of U/P solute ratios between critically ill and healthy cohorts.

**Figure 1:**
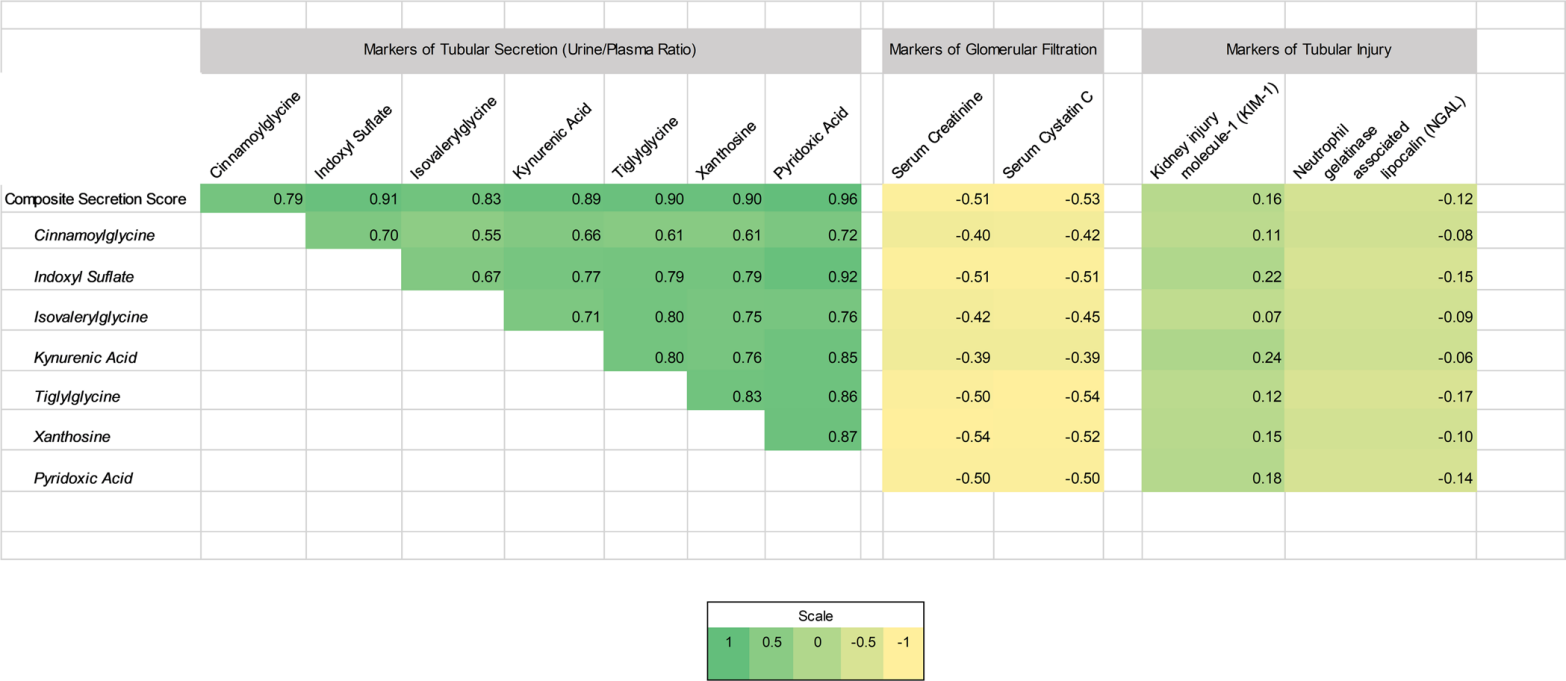
Relationships among markers of tubular secretion and markers of glomerular filtration. Spearman’s correlation matrix represents relationships among log-transformed U/P ratios of tubular secretory solutes in the CITRC (*n* = 170). Coefficients (*r*) are presented. Color intensity corresponds to the effect size (*r*). U/P, urine-to-plasma ratio; CITRC, Critical Illness Translational Research Cohort.

Secretory solute U/P ratios of 6 of 7 secretory solutes were on average 2–10 times lower in critically ill patients compared with healthy individuals after adjustment for age, sex, urine, and SCr levels. In particular, secretory ratios of isovalerylglycine and tiglylglycine differed most consistently between the cohorts (*P* < 0.001; Table 2). Secretory solute U/P ratios were variably correlated with each other (range of correlation *r* = 0.55–0.96; Figure 1). Each of the 7 secretory solutes was minimally to moderately correlated with SCr and cystatin C (range of correlation *r* = –0.39 to –0.54). We then computed the composite secretion score as the average of the 7 U/P ratios. The composite secretion score was highly correlated with each of the 7 individual secretory solutes (range of correlation *r* = 0.79–0.96). In contrast, the composite secretion score was inversely and modestly correlated with SCr and cystatin C (*r* = –0.51 and –0.53, respectively).

### Associations of baseline characteristics with U/P solute ratios.

**Figure 2:**
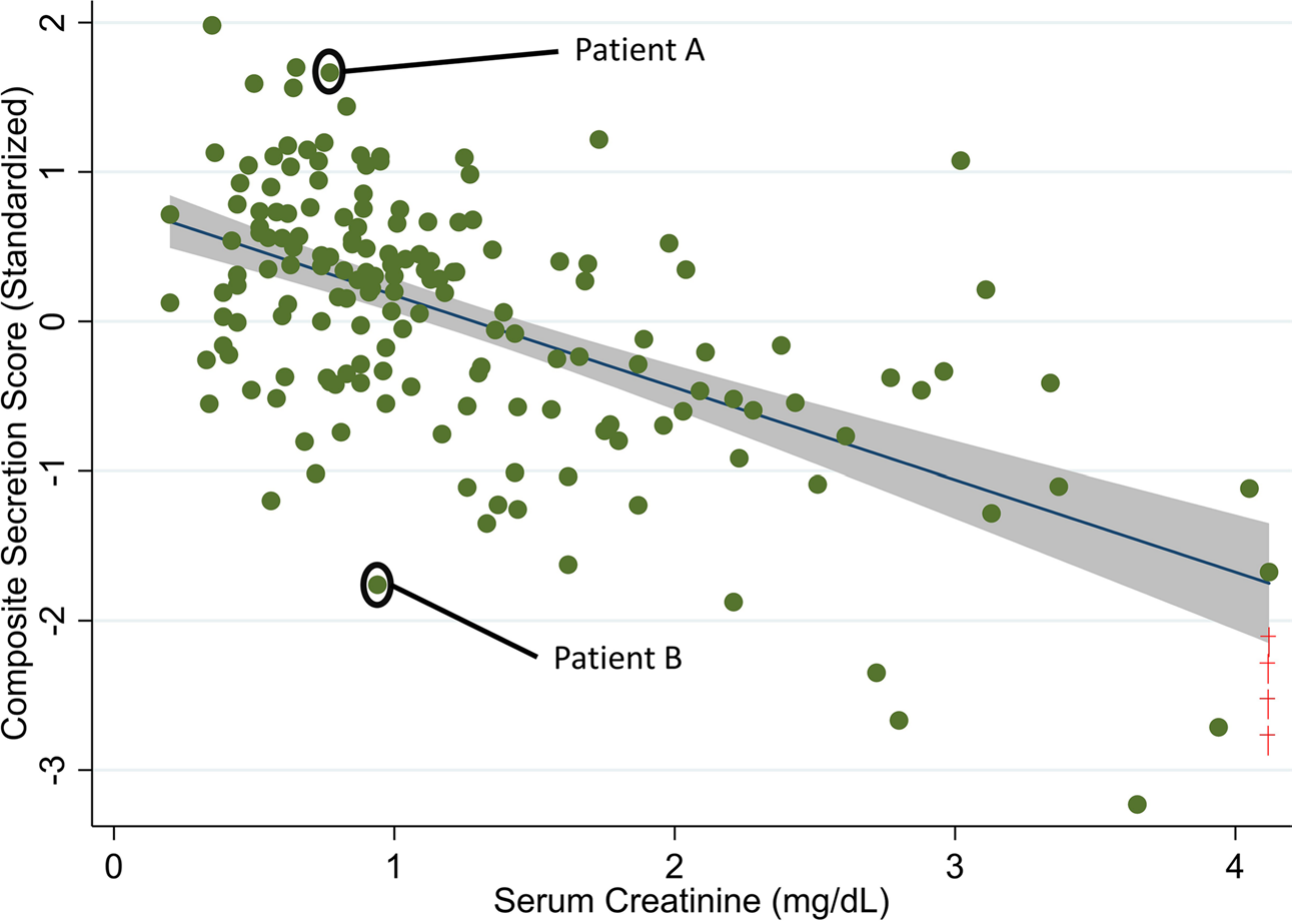
Visualization of correlations between ranges of composite secretion score and SCr measured at study enrollment in a critically ill population. The scatterplot allows for visual examination of the range, variability, and interindividual differences between the composite secretion score and SCr. To ease comparison of different tubular solutes, we standardized or rescaled solute measurements to have a mean of 0 and a SD of 1. We then computed the composite secretion score as the average of the 7 standardized U/P ratios. Red crosses at the right represent participants with extremely high SCr, who are displayed at an arbitrary maximum range value for graphic examination purposes; these participants are included in all statistical analyses using the true data value. Regression line is fit with 95% CIs. To demonstrate the interindividual variability in tubular secretion, we highlight 2 patients (patients A and B) with similar SCrs (approximately 1 mg/dL) but extremes of tubular secretion. Patient A is in the highest tertile of tubular secretion, whereas patient B is in the lowest tertile of tubular secretion.

Participants in the lowest tertile of the composite secretion score had a higher prevalence of AKI at study enrollment, were more likely to have a diagnosis of septic shock, and were more likely to require vasopressors compared with participants in the highest tertile (Table 1 and Supplemental Table 2; supplemental material available online with this article; https://doi.org/10.1172/jci.insight.145514DS1). SCr measured at study enrollment tended to be lower in CITRC participants in the highest tertile of the composite secretion score; yet, large interindividual variation relative to SCr was observed (Figure 2). For example, 25% of patients in the lowest tertile of the composite secretion score had a SCr concentration that was less than 1 mg/dL. Relatively low tubular secretion was also associated with the use of piperacillin-tazobactam but not other commonly used ICU medications (Supplemental Table 3).

### Associations between tubular solute U/P ratios and clinical outcomes.

The primary outcome of MAKE_28_ occurred in 50 (29%) participants, with a qualifying event of death in 11, requirement for dialysis in 5, and a doubling in SCr in 34. Higher U/P ratios of each secretory solute and the composite secretion score were associated with a lower risk of MAKE_28_ (Table 3 and Supplemental Figure 2). In fully adjusted models, each SD higher composite secretion score was associated with a 25% lower risk of MAKE_28_ (relative risk 0.75; 95% CI 0.62 to 0.91; *P* < 0.001). Individually, higher U/P ratios of isovalerylglycine and tiglylglycine were associated with the development of MAKE_28_ even after correction for multiple testing (relative risk = 0.72, 95% CI 0.57 to 0.91; and relative risk = 0.70, 95% CI 0.58 to 0.84, respectively). In sensitivity analyses, associations were not materially altered by adjusting for cystatin C instead of SCr or by adjusting for the ratio of urine to SCr (Supplemental Table 4 and Supplemental Table 5). The composite secretion score was also associated with doubling of SCr or dialysis within 7 days of study enrollment (Supplemental Table 6). Plasma concentrations of tubular solutes were associated with the development of MAKE_28_, suggesting renal retention is reflected by elevated plasma concentrations (Supplemental Table 7).

In sensitivity analyses, we stratified patients with AKI upon study enrollment into etiologies of AKI (prerenal and acute tubular necrosis [ATN]) based on the fractional excretion of filtered sodium. Among 45 patients with AKI upon study enrollment, 18 had prerenal AKI and 15 had ATN. The U/P ratio of tubular solutes was 2- to 3-fold higher in patients with prerenal AKI compared with ATN (Supplemental Table 8). We also compared U/P ratios and fractional excretions of tubular solutes. We found that both a higher U/P ratio and a higher fractional excretion of tubular solutes were associated with a lower risk of MAKE_28_. These findings provide reassurance that there was net secretion of each of these solutes (Supplemental Table 9).

In exploratory subgroup analyses, the size of associations between the composite secretion score and MAKE_28_ was statistically similar across strata defined by Sepsis-3 status and septic shock upon study enrollment. In contrast, the risk of MAKE_28_ was statistically different between patients with and without AKI upon study enrollment (*P* value for interaction 0.03). Patients with AKI upon study enrollment had a reduced risk for MAKE_28_, with higher composite secretion score; but in patients without AKI, there was no significant difference in MAKE_28_ outcomes based on the composite secretion score (Table 4).

### Comparisons of tubular solute U/P ratios and urinary biomarkers of tubular injury.

The composite secretion score was minimally correlated with urinary kidney injury molecule 1 (KIM-1; *r* = 0.16) and neutrophil gelatinase–associated lipocalin (NGAL; *r* = –0.12) concentrations at the time of study enrollment. NGAL concentrations were associated with higher risk of MAKE_28_ in fully adjusted models (relative risk, 1.10; 95% CI 1.02 to 1.18; *P* = 0.013), whereas KIM-1 concentrations were not associated with MAKE_28_ (Supplemental Table 10). We then tested whether tubular solute U/P ratios were associated with MAKE_28_ after accounting for urinary biomarkers of tubular injury. In models adjusted for urinary KIM-1 concentrations, each SD higher composite secretion score continued to be associated with a lower risk of MAKE_28_ (relative risk, 0.68; 95% CI 0.54 to 0.85; *P* = 0.001). We also found that the composite secretion score continued to be associated with a lower risk of MAKE_28_, after adjusting for urinary NGAL concentrations (relative risk, 0.79; 95% CI 0.66 to 0.94; *P* = 0.008; Supplemental Table 11).

## Discussion

We found lower U/P ratios of endogenous secretory solutes in critically ill patients compared with healthy control subjects. Among critically ill patients, higher U/P ratios of isovalerylglycine and tiglylglycine and a higher composite secretory score were associated with a lower risk of MAKE_28_, after controlling for demographics, ICU severity of illness, SCr, and urinary biomarkers (KIM-1 and NGAL). Our findings suggest that the measurement of proximal tubular secretion may provide additional diagnostic and prognostic information about kidney function in critical illness that complements measures of glomerular filtration (e.g., creatinine). Assessment of both secretion and filtration in the ICU could improve risk stratification of patient populations and improve phenotyping of AKI in the ICU. To our knowledge, this is the first study in critically ill patients to characterize tubular secretory function and determine the independent association with clinical outcomes.

U/P ratios of the secretory solutes in this study were markedly lower in patients with critical illness compared with healthy controls. This finding is consistent with knowledge that all of these solutes are putative substrates of basolateral organic anion transporters (OAT1 and OAT3) in the proximal tubule (14, 19). U/P ratios of isovalerylglycine and tiglylglycine differed most consistently between the critically ill and healthy populations in this study, and, in turn, these solutes were most strongly associated with the development of MAKE. It is possible that the strength of the association between isovalerylglycine and tiglylglycine and outcomes is due to differences in the affinity for specific basolateral transporters and differences in protein binding or cellular catabolism. For example, isovalerylglycine and tiglylglycine are glycine conjugates that are metabolites of fatty acids. These 2 solutes have been shown to be not only filtered but also highly secreted, and spot blood and urine measurements perform well to estimate tubular secretion correlate compared with 24-hour tubular clearances (20, 21).

We evaluated patients early after ICU admission to assess the decoupling that may have occurred between glomerular filtration and tubular secretion among a broad spectrum of SCrs. We found that the correlation of U/P ratios of tubular solutes and creatinine was equally poor in participants with and without AKI at study enrollment. We then looked at whether receipt of ICU medications that are known substrates of secretory OAT1/3 transporters in the proximal tubule may explain the discordance between SCr and tubular secretion. We found that receipt of piperacillin-tazobactam, but not other common ICU drugs, was more prevalent among patients with lower tubular secretion. We also found that in patients with the same creatinine concentration, U/P ratios of tubular secretion were associated with the longitudinal development of MAKE_28_. In the context of experimental and clinical evidence that proximal tubular epithelial cells are susceptible to injury in common ICU diagnoses, such as sepsis and shock (22, 23), our findings suggest that tubular secretion may be an early marker of worsening kidney function.

These results suggest a paradigm for evaluating kidney function in the ICU. The proximal tubules of the kidneys are particularly susceptible to injury due to sepsis, ischemia, and nephrotoxins. The ensuing reduction in the glomerular filtration rate (GFR) that follows tubular injury is governed by numerous kidney and host responses, including altering systemic and local hemodynamics, tubuloglomerular feedback, ongoing endothelial injury, and changes in vascular tone. Heterogeneity in these mechanisms across individuals likely contributes to the observed variation between estimated secretory clearance and glomerular filtration. Although previous research has demonstrated that sensitive markers such as NGAL and KIM-1 may potentially serve as a beacon for early tubular injury (24, 25), the current findings extend upon this work by revealing that the functional consequences of tubular injury are distinct from glomerular filtration, clinically important, and measurable. The specific reduction of secretory clearance may have direct clinical consequences because many endogenous secretory solutes are uremic toxins that are tightly bound to albumin and other large proteins and conversely are poorly eliminated through glomerular filtration. Studies have demonstrated associations between plasma concentrations of uremic toxins and risk of cardiovascular, skeletal, kidney, and cognitive impairment among patients with CKD and end-stage renal disease (26–29). Thus, a reduction in tubular secretion and subsequent retention of these uremic solutes could plausibly contribute to worsening ICU clinical outcomes.

This study includes several important limitations. First, we used spot U/P ratios of secretory solutes as a surrogate for timed clearances. However, timed urinary clearances, as performed in stable outpatients, require steady-state plasma concentrations and are less accurate when kidney dysfunction is evolving (30). Moreover, in outpatients we have shown that supervised 10-hour timed urine collections and spot U/P measurements in the same people on the same day corresponded very closely (23). Second, laboratory measurement error in determining solute concentrations in blood and urine may have falsely exaggerated the lack of correlation between tubular secretion relative to creatinine. However, laboratory precision was generally high and such misclassification would be expected to be nondifferential and bias associations to the null. In contrast, we found that tubular secretion was strongly associated with the development of MAKE_28_. Third, we did not perform goal standard measurements of filtration using exogenous markers, such as iothalamate, but instead relied on clinically used markers, such as creatinine, and, to a lesser extent, cystatin C. Fourth, the small sample size and single study center hinder the generalizability of the results. However, it was reassuring that the point estimate for each secretory solute consistently demonstrated a decreased risk for MAKE with an increase in secretory solute U/P ratios. Future work in larger cohorts may allow distinguishing differences in risk of clinical outcomes between individual tubular secretory solutes.

This study has several strengths. Exposure variables and outcomes were collected prospectively by trained research coordinators. We developed a precise targeted LC-MS/MS assay for endogenous secretory solutes based on labeled internal standards and external calibrators, including standardization to purified chemical compounds analyzed by quantitative nuclear magnetic resonance. To our knowledge, this is the first study in a critically ill population to demonstrate associations of estimated secretory solute clearance with clinical outcomes. Spot samples, instead of timed urine and plasma samples, may ease the translation of our findings to clinical care, because timed urine collections are often challenging to complete in the ICU (31). Associations between U/P ratio of secretory solutes and clinical outcomes were independent of demographics, ICU severity of illness, SCr, and urinary biomarkers, suggesting tubular secretion is an independent kidney function that has prognostic implications. It is also worth highlighting that plasma secretory solute measurements were also independently associated with MAKE_28_ outcomes and would be a more practical measure to estimate secretory clearance, instead of spot blood and urine samples. Future work in larger sample sizes with timed urine collections could compare the correlation of plasma secretory solute measurements and U/P secretory solute ratios with the gold standard of timed clearances.

In conclusion, this study demonstrates the first characterization of proximal tubular secretion as an independent marker of kidney function in a longitudinal cohort of critically ill participants. These data motivate additional investigation of tubular secretion as a measure of kidney function, with potential applications in early identification of AKI complications and outcomes in critical illness and improving dosing of ICU drugs that undergo proximal tubular secretion.

## Methods

### CITRC study population.

CITRC is a prospective cohort study of ICU patients at high risk of organ dysfunction. CITRC began prospective enrollment of patients from surgical and medical ICUs at Harborview Medical Center in Seattle, Washington, beginning in 2015. Eligibility included meeting at least 2 of 4 criteria for the systemic inflammatory response syndrome; admission to a medical, surgical, or cardiac ICU; and not receiving any form of renal replacement therapy. Patients were enrolled within 24 hours of ICU admission. Exclusion criteria included inability to provide informed consent, non–English-speaking, metastatic cancer, severe immunosuppression, vulnerability, and do-not-resuscitate or comfort care orders within the first 24 hours of ICU admission. For this ancillary study, we selected the first 170 CITRC participants who had available spot urine and blood samples within 24 hours of ICU admission. All urine samples were collected from patients with an indwelling urinary catheter. Supplemental Figure 1 provides a flow diagram of participant enrollment.

### HKS population.

For comparison, we obtained blood and spot urine samples from 70 participants in the HKS, a study of individuals who were free of clinically apparent kidney disease, defined by an estimated GFR of greater than or equal to 90 mL/min/1.73m^2^ and a urine albumin-to-creatinine ratio of less than 30 mg/g. The HKS recruited persons from general and family medicine clinics throughout the University of Washington system between 2012 and 2016.

### Estimates of secretory solute clearance.

We originally selected 15 candidate secretory solutes based on extensive literature review and discussion with colleagues in this field. Literature review criteria included at least 1 of the following: specificity for OAT1/3 transporters; a high degree of protein-binding, suggesting minimal glomerular filtration and/or kidney clearances that substantially exceed that of creatinine; a primarily filtered substance; and potential for accurate measurement by our LC-MS/MS methods (28, 32, 33). Three solutes could not be measured reliably in our laboratory. An additional 4 solutes exhibited unacceptably high diurnal variation in a study of 26 healthy volunteers. Given the small sample size of this study, we decided to further exclude 1 solute (p-cresol sulfate) due to its very high correlation with indoxyl sulfate, which was included in the analysis. We then developed a targeted LC-MS/MS assay for these solutes in plasma and urine using labeled internal standards and external calibration (26). We performed empiric protein-binding studies in our laboratory from samples of patients with critical illness, which revealed lower protein-binding percentages than previously reported for 3 solutes (isovalerylglycine, tiglylglycine, and xanthosine) among critically ill adults (Supplemental Table 1). However, we retained these solutes in the analyses because their kidney clearances greatly exceeded estimated GFR, suggesting tubular secretion as the major kidney pathway of elimination. Additional methods can be found in Supplemental Methods.

We measured total plasma concentrations of secretory solutes using protein precipitation, solid phase extraction, and LC-MS/MS and spot urine concentrations using solid phase extraction and LC-MS/MS. Calibration was achieved using a single-point calibration approach to correct for potential drift that may be caused by changes in reagents, calibrator lots, equipment, or settings. The peak areas of each solute were normalized to peak areas of labeled internal standards added to each well. Peak area ratios were then standardized to single-point calibrators (mean of 5 replicates run performed on each plate). We have previously determined accurate concentrations of each solute in the single-point calibrators (pooled human serum and urine) by standard addition of solutions of pure compounds analyzed by quantitative nuclear magnetic resonance. Laboratory coefficients of variation were generally low (Supplemental Table 1). We have found no changes in the concentrations of these solutes with up to 3 freeze-thaw cycles.

We estimated the kidney clearance of each solute as *U_X_*/*P_X_*, where *U_X_* represents the spot urine concentration of solute X and *P_X_* represents the plasma concentration. To account for potential differences in urine concentrations across individuals, we adjusted analyses for the urinary creatinine concentration.

### Urinary biomarker measurements.

In urine samples, we measured KIM-1 and NGAL using a multiplex assay (Meso Scale Discovery). Biomarkers were measured in 1 batch and the intraplate coefficient of variation for KIM-1 was 15% and for NGAL was 13%.

### Measurement of outcomes.

CITRC research coordinators identified the initiation of inpatient dialysis and death by chart review, inpatient visits, and telephone callbacks to patients. Outcomes were adjudicated until 28 days after ICU admission. The primary outcome MAKE_28_ was defined as (a) doubling of SCr concentration from the creatinine measurement at CITRC study enrollment, (b) receipt of any renal replacement therapy, or (c) death. We selected MAKE_28_ to consider the breadth of clinical consequences in the ICU and to account for issues of competing risk due to death (34).

### Measurement of other study data.

AKI at study enrollment was defined as a greater than or equal to 0.3 mg/dL and/or greater than or equal to 50% increase in SCr measurement at the time of study enrollment compared with the baseline SCr value measured prior to study enrollment or a decrease in urine output (35). We determined the baseline creatinine value using a hierarchical approach in which creatinine values obtained during the year prior to the index hospitalization were given priority over in-hospital measurements obtained before study enrollment. We measured creatinine in blood and urine samples and cystatin C in blood samples collected from participants at study enrollment in CITRC. Serum and urine creatinine concentrations were measured using the modified Jaffe method, and plasma cystatin C concentrations were measured using an immunoturbidimetric assay (Gentian AS) on a Beckman DXC Unicell clinical analyzer. Prevalent diabetes was defined by participant self-report of diabetes or the use of insulin. CKD stage III or greater as a GFR less than 60 mL/min per 1.73 m^2^ was calculated by the Chronic Kidney Disease Epidemiology Collaboration equation (36) using a patient’s baseline creatinine prior to hospitalization or based on chart diagnosis. We defined septic shock as meeting Sepsis-3 criteria and hypotension (37). Mechanical ventilation and vasopressors were defined as any time during ICU admission requiring these therapies.

### Statistics.

To facilitate presentation of participant characteristics and provide a single metric for summarizing associations, we computed a composite secretion score as the average of the individual U/P ratios. To account for right-skewed data, we log-transformed the individual solute clearances. To ease the comparison of different solutes, we standardized or rescaled solute measurements to have a mean of 0 and a SD of 1. We then computed the composite secretion score as the average of the 7 U/P ratios. We present joint distributions of secretory solutes using Spearman’s correlation coefficients, and we used multiple linear regression to compare secretory solute ratios between HKS and CITRC cohorts.

We performed relative risk regression using a multivariate generalized linear model to test for associations between MAKE_28_ (dependent variable) and the standardized U/P ratio of each secretory solute (independent variable). We used a Gaussian model and robust standard error estimates. We present univariate and multivariate associations between secretory solute ratios and kidney events as relative risks per 1 SD difference in U/P ratios.

We selected adjustment variables a priori on the basis of biologic plausibility and prior literature, suggesting these variables may confound associations between U/P ratios of secretory solutes and clinical outcomes. The first adjusted model included age, sex, Black race, urine albumin-to-creatinine concentrations (to account for differences in urine volume), AKI status on study enrollment, and SOFA excluding the renal component (38). The SOFA score was based on maximal variables obtained during the first day of ICU admission. The second model added adjustment for SCrs or cystatin C concentrations measured at CITRC study enrollment. Because we tested 8 associations between secretory solutes (7 secretory solutes and the composite secretion score) and kidney events, we chose the conservative estimate of a Bonferroni’s corrected *P* value cutoff of 0.05/8 = 0.00625 to declare significance. We also performed subgroup analyses to explore whether the size of associations between the composite secretion score and MAKE_28_ differed by AKI status on study enrollment, shock, and Sepsis-3. All analyses were performed using Stata release 15.1 software (StataCorp).

### Study approval.

CITRC samples were collected as approved by the University of Washington Institutional Review Board protocol 1389. Informed consent was obtained from patients or their proxies. All human and animal studies have been approved by the appropriate ethics committee and have therefore been performed in accordance with the ethical standards laid down in the 1964 Declaration of Helsinki and its later amendment.

## Author contributions

PKB, MMW, and BRK conceived the study and design. PKB, MMW, BRK, XYC, NAS, JR, EDS, JH, and ANH acquired, analyzed, or interpreted the data. PKB drafted the manuscript. PKB, MMW, BRK, XYC, NAS, JR, EDS, JH, and ANH provided critical revision of the manuscript for important intellectual content. PKB and BRK provided statistical analysis. MMW and BRK supervised the study.

## Supplementary Material

Supplemental data

Trial reporting checklists

ICMJE disclosure forms

## Figures and Tables

**Table 1:**
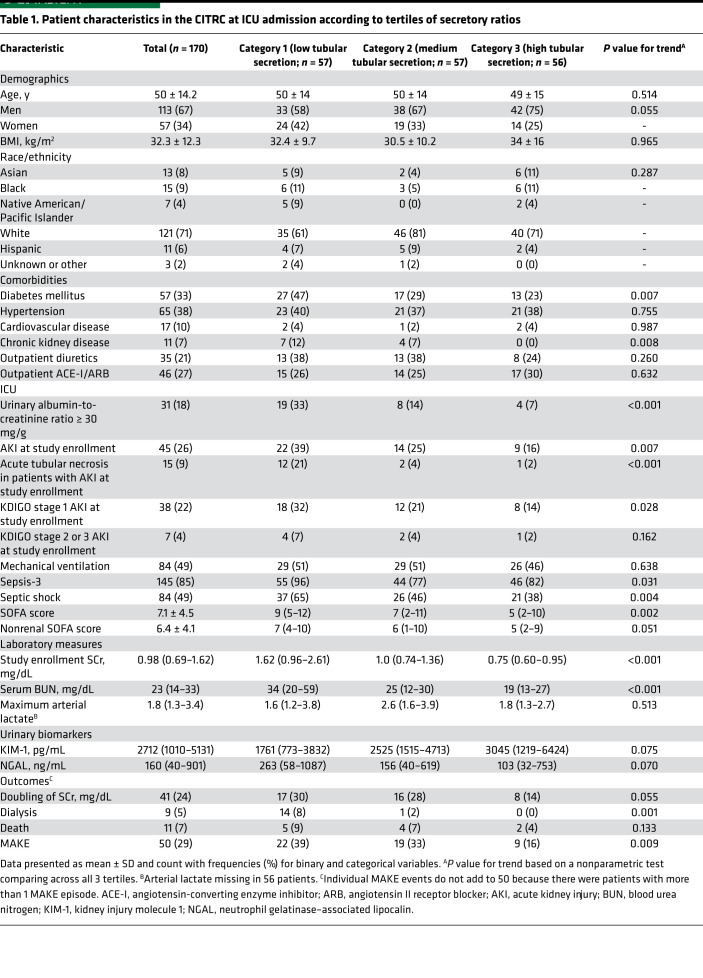
Patient characteristics in the CITRC at ICU admission according to tertiles of secretory ratios

**Table 2:**
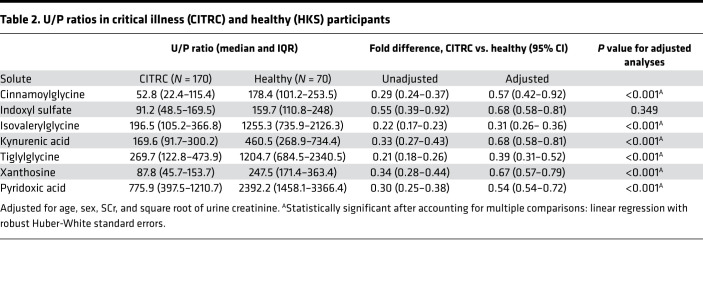
U/P ratios in critical illness (CITRC) and healthy (HKS) participants

**Table 3:**
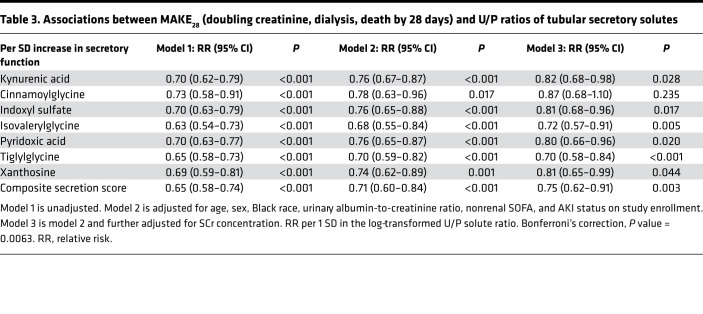
Associations between MAKE_28_ (doubling creatinine, dialysis, death by 28 days) and U/P ratios of tubular secretory solutes

**Table 4:**
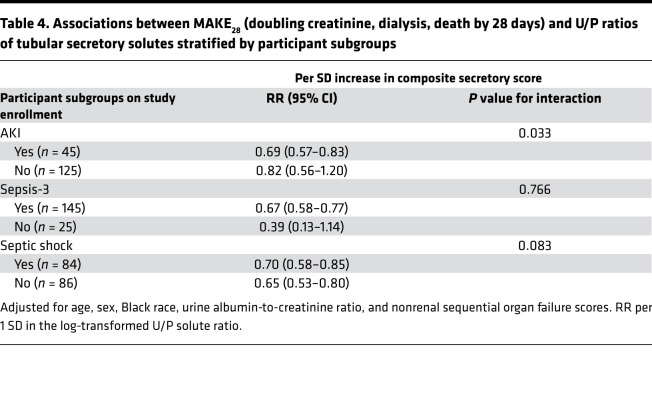
Associations between MAKE_28_ (doubling creatinine, dialysis, death by 28 days) and U/P ratios of tubular secretory solutes stratified by participant subgroups

## Version 1 04/22/2021

In-Press Preview

## Version 2 05/24/2021

Electronic publication
