# Supplementary material for: Assessment of kidney proximal tubular secretion in critical illness
Source: JCI Insight. 2021 May 24;6(10):e145514. doi: 10.1172/jci.insight.145514 (PMC8262320; doi:10.1172/jci.insight.145514)

ONLINE SUPPLENTARY MATERIALS

Title: Assessment of Kidney Proximal Tubular Secretion in Critical Illness

Authors: Bhatraju PK, Chai XY, Sathe NA. et al.

TABLE OF CONTENTS

Additional eMethods

Table S1. Laboratory characteristics and variability of secretory solutes

Table S2. Distribution of U/P ratio of tubular solutes at study enrollment in CITRC among patients who do not develop AKI, patients with AKI at study enrollment and patients who develop a doubling of serum creatinine during the study

Table S3. Distribution of secreted ICU medications between tertiles of tubular secretion

Table S4. Associations between MAKE (doubling creatinine, dialysis, death by 28 days) and urine/plasma ratios of tubular secretory solutes adjusted for either serum creatinine or serum cystatin C

Table S5. Associations between MAKE (doubling creatinine, dialysis, death by 28 days) and urine/plasma ratios of tubular secretory solutes adjusted for either serum creatinine or urine/serum creatinine concentrations

Table S6. Associations between doubling serum creatinine and dialysis by 7 days and urine/plasma ratios of tubular secretory solutes

Table S7. Associations between MAKE (doubling creatinine, dialysis, death by 28 days) and plasma tubular secretory solutes

Table S8. Distribution of U/P ratio of tubular solutes at study enrollment in CITRC among patients with pre-renal AKI and acute tubular necrosis based on FENa among 45 patients with AKI at study enrollment

Table S9. Associations between MAKE (doubling creatinine, dialysis, death by 28 days) and urine/plasma ratios or fractional excretion of tubular secretory solutes

Table S10. Associations between MAKE (doubling creatinine, dialysis, death by 28 days) and KIM-1 and NGAL

Table S11. Associations between MAKE (doubling creatinine, dialysis, death by 28 days) and urine/plasma ratios of tubular secretory solutes adjusted for either KIM-1 and NGAL

Figure S1. Flow of participant enrollment and study inclusion in CITRC

Figure S2. Markers of tubular secretion associated with risk of the composite of major adverse kidney events, MAKE (doubling of creatinine, dialysis and death) within 28 days of ICU admission

Additional Methods

Serum solute measurements were of total concentration: samples were extracted using formic acid/acetonitrile as part of the protein precipitation step of liquid chromatography-MS/MS assays, which removes all bound solutes from serum/plasma proteins. Residual protein remnants were removed by filtration and evaporation and then, discarded. The extracted serum/plasma was reconstituted in water for subsequent MS/MS quantification. Spot urine solutes were quantified using similar methods without the protein precipitation steps. Isotope dilution with stable isotope-labeled internal standards and external calibration materials maximized precision. Fragment ion ratios were monitored for specificity. Matrix effects were minimal at low concentrations of analytes for urine, serum and plasma samples.

Table S1. Laboratory characteristics and variability of secretory solutes.[a]

	Molecular weight (g/mol)	Intra-assay CV plasma (%)	Inter-assay CV plasma (%)	Intra-assay CV urine (%)	Inter-assay CV urine (%)	Protein binding in critical illness
Cinnamoylglycine	205	4.5	5.4	4.9	4.5	80 ±20%
Indoxyl sulfate	213	4.3	6.0	6.1	9.4	89 ±5%
Isovalerylglycine	159	7.1	7.3	5.4	5.9	28 ±17%
Kynurenic acid	189	4.1	5.5	5.6	8.6	95 ±2%
Pyridoxic acid	183	3.4	4.7	5.7	5.8	64 ±13%
Tiglylglycine	157	7.0	14.7	6.0	5.5	25 ±24%
Xanthosine	284	11.1	14.5	9.8	10.1	27 ±6%

[a] CV: coefficient of variation.

Table S2. Distribution of U/P ratio of tubular solutes at study enrollment in CITRC among patients who do not develop AKI, patients with AKI at study enrollment and patients who develop a doubling of serum creatinine during the study

	**Urine to plasma ratio (Median and interquartile range) for CITRC patients**			***p-value* for trend**[a]
**Solute**	**No AKI at enrollment No doubling of serum creatinine during study (n=98)**	**AKI at Enrollment No doubling of serum creatinine during study (n=31)***	**Doubling of Serum Creatinine during study (n=41)***	
Cinnamoylglycine	57.1 (31.2 – 115.4)	49.8 (18.1 – 125.5)	36.7 (14.8 – 75.5)	0.013
Indoxyl Sulfate	102.3 (59.3 – 184.5)	72.5 (38.9 – 122.4)	67.9 (30.8 – 157.3)	0.013
Isovalerylglycine	234.6 (142.7 – 428.1)	161.6 (104.9 – 417.4)	132.4 (75.1 – 251.6)	0.001
Kynurenic acid	182.7 (106.6 – 316.7)	154.1 (90.1 – 241.5)	142.5 (41.7 – 220.2)	0.021
Tiglylglycine	287.2 (152.2 – 577.6)	286.9 (130.2 – 466.5)	175.7 (79.3 – 354.0)	0.007
Xanthosine	103.2 (53.7 – 167.9)	74.5 (41.6 – 155.2)	62.2 (34.1 – 118.4)	0.007
Pyridoxic acid	909.9 (419.6 – 1377.5)	677.1 (414.1 – 1006.7)	559.7 (249.3 – 931.6)	0.005

[a] *p-value* for trend based on a non-parametric test comparing across all three categories
14 patients had AKI at study enrollment and also developed a doubling of creatinine during hospitalization. These patients are including in the doubling of creatinine category.

Table S3. Distribution of secreted ICU medications among tertiles of tubular secretion

Medications[a]	Category 1 (Low Tubular Secretion; n=57)	Category 2 (Medium Tubular Secretion; n=57)	Category 3 (High Tubular Secretion; n=56)	p-value for trend[b]
Cefepime	3 (5)	2 (3)	2 (3)	0.651
Fentanyl	2 (3)	1 (2)	4 (7)	0.335
Midazolam	15 (26)	17 (30)	10 (18)	0.301
Piperacillin-tazobactam	33 (58)	24 (42)	12 (21)	<0.001
Furosemide	6 (11)	1 (2)	6 (11)	0.978
Ranitidine	6 (11)	3 (5)	2 (4)	0.133
Pantoprazole	20 (35)	30 (53)	15 (27)	0.373

[a] We selected specific drugs based on their frequency of use in the ICU and plausibility that secretory clearance is a primary or contributing mechanism of elimination.

[b] p-value for trend based on a non-parametric test comparing across all three tertiles

Table S4. Associations between MAKE (doubling creatinine, dialysis, death by 28 days) and urine/plasma ratios of tubular secretory solutes adjusted for either serum creatinine or serum cystatin C

**Per SD increase in secretory function**	**Model 1 and Serum Creatinine: RR (95% CI)**	***p***	**Model 1 and Serum Cystatin C: RR (95% CI)**	***P***
Kynurenic Acid	0.82 (0.68 – 0.98)	0.028	0.81 (0.69 – 0.94)	0.005
Cinnamoylglycine	0.87 (0.68 – 1.10)	0.235	0.84 (0.67 – 1.05)	0.133
Indoxylsuflate	0.81 (0.68 – 0.96)	0.017	0.80 (0.67 – 0.97)	0.019
Isovalerylglycine	0.72 (0.57 – 0.91)	0.005	0.71 (0.57 – 0.89)	0.003
Pyridoxic Acid	0.80 (0.66 – 0.96)	0.020	0.79 (0.65 – 0.96)	0.015
Tiglylglycine	0.70 (0.58 – 0.84)	<0.001	0.71 (0.59 – 0.84)	<0.001
Xanthosine	0.81 (0.65 – 0.99)	0.044	0.79 (0.65 – 0.96)	0.018
**Composite Secretion Score**	**0.75 (0.62 – 0.91)**	**0.003**	**0.75 (0.62 – 0.89)**	**0.002**

Model 1 adjusted for age, gender, black race, urine albumin-creatinine ratio, non-renal sequential organ failure scores (SOFA), AKI on study enrollment

RR per 1 standard deviation in the log-transformed urine/plasma solute ratio

Table S5. Associations between MAKE (doubling creatinine, dialysis, death by 28 days) and urine/plasma ratios of tubular secretory solutes adjusted for either serum creatinine or urine to serum creatinine ratio

Per SD increase in secretory function	Model 1 and Serum Creatinine: RR (95% CI)	***p***	Model 1* and Urine/Serum (U/S ratio) Creatinine: RR (95% CI)	***P***
Composite Secretion Score	0.75 (0.62 – 0.91)	0.003	0.67 (0.53 – 0.83)	<0.001

Model 1 adjusted for age, gender, black race, urine albumin to creatinine ratio, AKI on study enrollment, non-renal sequential organ failure scores (SOFA)

Model 2 adjusted for age, gender, black race, AKI on study enrollment, non-renal sequential organ failure scores (SOFA) and U/S creatinine ratio

RR per 1 standard deviation in the log-transformed urine/plasma solute ratio

Table S6. Associations between doubling serum creatinine and dialysis by 7 days and urine/plasma ratios of tubular secretory solutes

**Per SD increase in secretory function**	**Model 1: RR (95% CI)**	***p***
Composite Secretion Score	0.59 (0.47 – 0.73)	<0.001

Model 1 adjusted for age, gender, black race, urine albumin to creatinine ratio, non-renal sequential organ failure scores (SOFA), AKI on study enrollment, serum creatinine

RR per 1 standard deviation in the log-transformed urine/plasma solute ratio

Table S7. Associations between MAKE (doubling creatinine, dialysis, death by 28 days) and U/P ratios and plasma tubular secretory solutes

Per SD increase in secretory function	Urine/Plasma ratios of tubular secretory solutes RR (95% CI)	*p*	Negative of plasma tubular solute solutes[a] RR (95% CI)	*p*
Kynurenic Acid	0.82 (0.68 – 0.98)	0.028	0.52 (0.40 – 0.67)	<0.001
Cinnamoylglycine	0.87 (0.68 – 1.10)	0.235	0.76 (0.63 – 0.93)	0.007
Indoxylsuflate	0.81 (0.68 – 0.96)	0.017	0.76 (0.57 – 1.00)	0.053
Isovalerylglycine	0.72 (0.57 – 0.91)	0.005	0.75 (0.63 – 0.90)	0.002
Pyridoxic Acid	0.80 (0.66 – 0.96)	0.020	0.89 (0.65 – 1.20)	0.438
Tiglylglycine	0.70 (0.58 – 0.84)	<0.001	0.76 (0.59 – 0.99)	0.041
Xanthosine	0.81 (0.65 – 0.99)	0.044	0.83 (0.51 – 1.35)	0.459
**Composite Secretion Score**	**0.75 (0.62 – 0.91)**	**0.003**	**0.51 (0.33 – 0.77)**	**0.002**

Models adjusted for age, gender, black race, urine albumin to creatinine ratio, non-renal sequential organ failure scores (SOFA), AKI on study enrollment, serum creatinine

[a] Exposure is negative to allow comparison of relative risks (RR)

RR per 1 standard deviation in the log-transformed plasma solute concentration

Table S8. Distribution of U/P ratio of tubular solutes at study enrollment in CITRC among patients with pre-renal AKI and acute tubular necrosis based on FENa among 45 patients with AKI at study enrollment

**Tubular Solutes**	**Urine to plasma ratio (Median and interquartile range) for CITRC patients**	
	**Pre-renal AKI (n=18)**	**Acute Tubular Necrosis AKI (n=15)**
Kynurenic acid	178 (150 – 300)	90 (37 – 133)
Indoxyl Sulfate	96 (62 – 152)	40 (21 – 73)
Isovalerylglycine	303 (169 – 500)	100 (53 – 144)
Cinnamoylglycine	105 (50 – 176)	24 (14 – 40)
Tiglylglycine	376 (277 – 474)	116 (63 – 213)
Xanthosine	98 (50 – 199)	42 (29 – 71)
Pyridoxic acid	794 (599 – 1190)	398 (178 – 683)

Table S9. Associations between MAKE (doubling creatinine, dialysis, death by 28 days) and urine/plasma ratios or fractional excretion of tubular secretory solutes

**Per SD increase in secretory function**	**Using U/P ratio: RR (95% CI)**	***p***	**Using fractional excretion: RR (95% CI)**	***P***
Kynurenic Acid	0.76 (0.65 – 0.88)	<0.001	0.78 (0.62 – 0.99)	0.042
Cinnamoylglycine	0.77 (0.63 – 0.95)	0.012	0.94 (0.75 – 1.18)	0.606
Indoxylsuflate	0.74 (0.63 – 0.88)	<0.001	0.74 (0.60 – 0.93)	0.008
Isovalerylglycine	0.68 (0.54 – 0.84)	<0.001	0.76 (0.61 – 0.96)	0.019
Pyridoxic Acid	0.75 (0.64 – 0.87)	<0.001	0.77 (0.62 – 0.96)	0.018
Tiglylglycine	0.69 (0.59 – 0.82)	<0.001	0.67 (0.58 – 0.78)	<0.001
Xanthosine	0.75 (0.62 – 0.90)	0.002	0.85 (0.52 – 1.40)	0.523
**Composite Secretion Score**	**0.70 (0.59 – 0.84)**	**<0.001**	**0.63 (0.46 – 0.87)**	**0.005**

Models adjusted for age, gender, black race, urine albumin to creatinine ratio, non-renal sequential organ failure scores (SOFA), AKI on study enrollment, serum creatinine

RR per 1 standard deviation in the log-transformed plasma solute concentration

Table S10. Associations between MAKE (doubling creatinine, dialysis, death by 28 days) and KIM-1 and NGAL

Per doubling increase in urinary biomarker	Model 1: RR (95% CI)	*p*	Model 2: RR (95% CI)	*P*
KIM-1	1.10 (0.96 – 1.27)	0.168	1.12 (0.97 – 1.31)	0.127
NGAL	1.10 (1.03 – 1.19)	0.007	1.10 (1.02 – 1.18)	0.013

Model 1 is adjusted for age, gender, black race, urine albumin-creatinine ratio, non-renal sequential organ failure scores (SOFA)
Model 3 is Model 1 and further adjusted for SCr

RR per 1 standard deviation in the $\log_{(2)}$ transformed KIM-1 and NGAL

Table S11. Associations between MAKE (doubling creatinine, dialysis, death by 28 days) and urine/plasma ratios of tubular secretory solutes adjusted for either KIM-1 and NGAL

**Per SD increase in secretory function**	**Model 1 and KIM-1: RR (95% CI)**	***p***	**Model 1 and NGAL: RR (95% CI)**	***P***
Composite Secretion Score	0.68 (0.54 – 0.85)	0.001	0.79 (0.66 – 0.94)	0.008

Model 1 adjusted for age, gender, black race, urine albumin to creatinine ratio, non-renal sequential organ failure scores (SOFA), AKI on study enrollment, serum creatinine

RR per 1 standard deviation in the log-transformed urine/plasma solute ratio

Figure S1. Flow of participant enrollment and study inclusion in CITRC

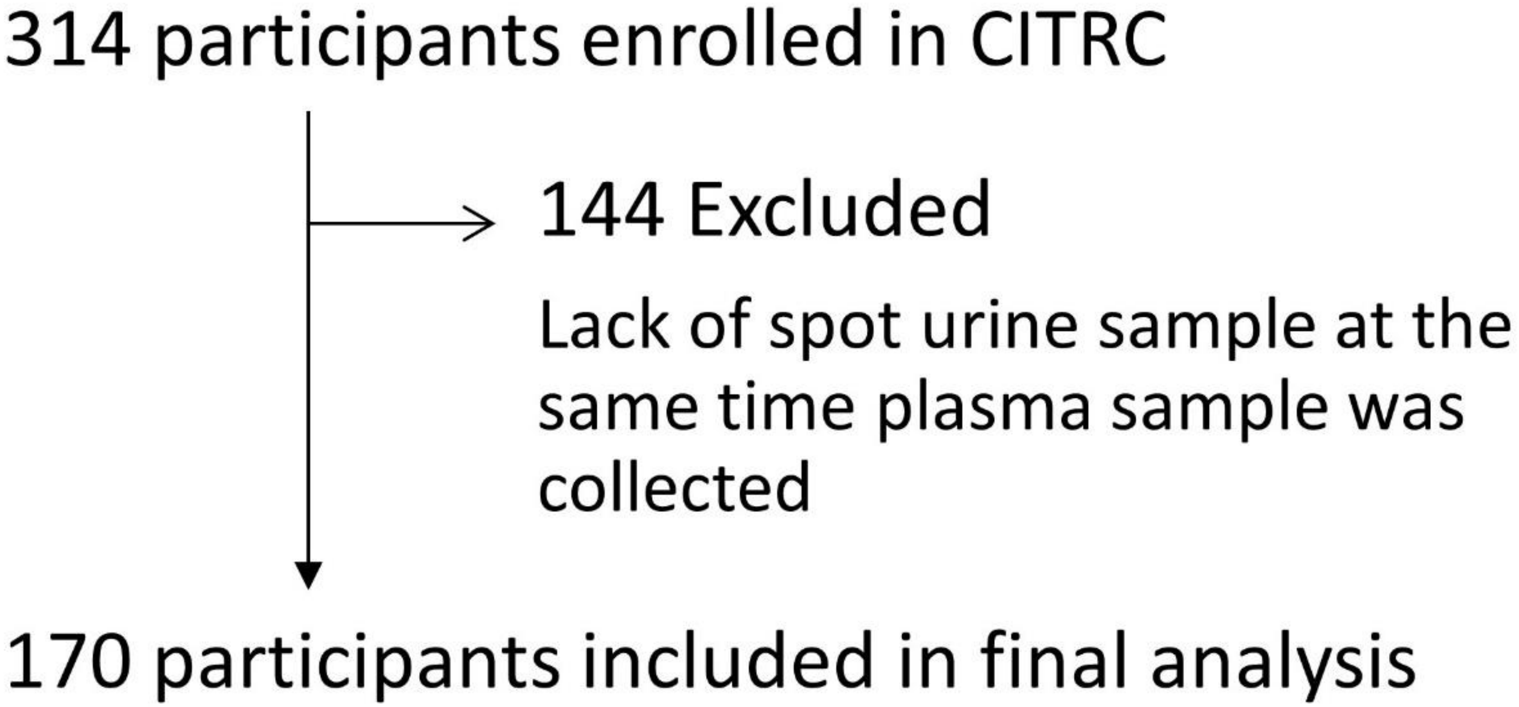

Figure S2. Markers of tubular secretion associated with risk of the composite of major adverse kidney events, MAKE (doubling of creatinine, dialysis and death) within 28 days of ICU admission. Markers of tubular secretion and 28-day risk of MAKE in the CITRC cohort (n=170). The effect is shown as a relative risk per one standard deviation change in the log-transformed urine/plasma ratio of each marker of tubular secretion. The composite secretion score is the average of each of the seven secretory solutes. Models are controlled for age, gender, black race, urine creatinine, sequential organ failure score (SOFA) excluding renal component and plasma creatinine concentrations. Asterisk * highlight *p-values* that are significant based on a conservative Bonferroni correction adjustment for multiple hypotheses *p*=0.0063.

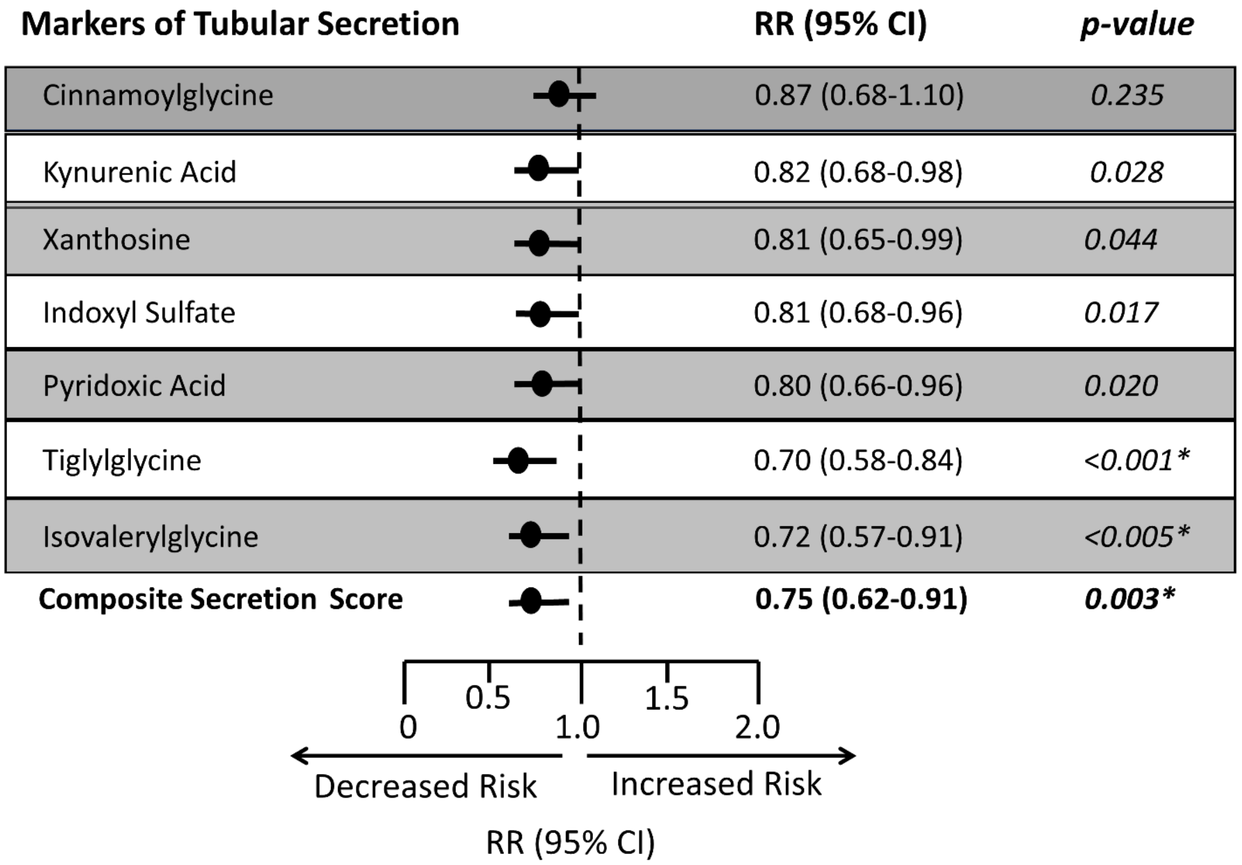

Supplement: Supplemental data [file jciinsight-6-145514-s256.pdf]
