# Supplementary material for: Assessment of kidney proximal tubular secretion in critical illness
Source: JCI Insight. 2021 May 24;6(10):e145514. doi: 10.1172/jci.insight.145514 (PMC8262320; doi:10.1172/jci.insight.145514)
Supplement: Trial reporting checklists [file jciinsight-6-145514-s257.pdf]

STROBE Statement—Checklist of items that should be included in reports of ***cohort studies***

	Item No	Recommendation
**Title and abstract**	1	(*a*) Indicate the study's design with a commonly used term in the title or the abstract<br>Abstract – cohort study of 170 ICU participants and 70 adults
		(*b*) Provide in the abstract an informative and balanced summary of what was done and what was found<br>We collected matched blood and spot urine samples from 170 ICU patients and from a comparison group of 70 adults with normal kidney function. We measured seven endogenously produced secretory solutes using liquid chromatography-tandem mass spectrometry. We computed a composite secretion score incorporating all seven solutes, and evaluated associations with 28-day major adverse kidney events (MAKE28), defined as doubling of SCr, dialysis dependence, or death.<br><br>The urine/plasma ratio of six of seven secretory solutes were lower in critically ill patients compared with normal individuals after adjustment for SCr. The composite secretion score was moderately correlated with SCr and cystatin C (r = -0.51 and r = -0.53, respectively). Each standard deviation higher composite secretion score was associated with a 25% lower risk of MAKE28 (95% CI 9% - 38% lower) independent of severity of illness, SCr and tubular injury markers. Higher urine to plasma ratios of individual secretory solutes isovalerylglycine and tiglylglycine were associated with MAKE28 after accounting for multiple testing.
**Introduction**		
Background/rationale	2	Explain the scientific background and rationale for the investigation being reported<br>Acute kidney injury (AKI) is the most common form of organ dysfunction in critically ill patients and is associated with prolonged hospitalization, requirement for acute dialysis, persistent kidney dysfunction, and death (1–3). Current definitions of AKI are based on incremental changes in SCr (4). However, prognosis and recovery patterns in AKI vary substantially across individuals with similar creatinine measurements, suggesting incomplete assessment of kidney function by estimates of glomerular filtration alone(5–8).<br><br>Most underlying causes of AKI, including sepsis, ischemia-reperfusion, hypoxemia, endogenous toxins, and nephrotoxic medications, involve injury to proximal tubular epithelial cells of the kidneys(9–12). The proximal tubules reabsorb filtered substances, synthesize vital hormones, maintain salt-water and acid-base homeostasis, and secrete retained organic solutes and drugs directly into the urine. Tubular secretory clearance is essential for eliminating protein-bound substances that cannot be filtered, many of which are uremic toxins that are linked with cognitive, skeletal muscle, and cardiac impairments(13–15). Tubular secretion is also the primary kidney mechanism for eliminating hundreds of commonly administered ICU medications, including antibiotics (cephalosporins, fluoroquinolones, aminoglycosides), sedatives (propofol, benzodiazepines), and diuretics(16–18). Creating more inclusive measurements of kidney function that include tubular secretory clearance could enable more precise ICU drug dosing strategies and improve prognostic assessment.
Objectives	3	State specific objectives, including any prespecified hypotheses<br>We hypothesized that estimates of proximal tubular secretory clearance would provide additional information about kidney function that is not captured by SCr levels alone.
**Methods**		
Study design	4	Present key elements of study design early in the paper<br>Study Populations<br><br>**The Critical Illness Translational Research Cohort (CITRC).** CITRC is a prospective cohort study of ICU patients at high-risk of organ dysfunction. CITRC began prospective enrollment of patients from surgical and medical ICUs at Harborview Medical Center in Seattle WA beginning in 2015. Eligibility included meeting at least two of four criteria for the systemic inflammatory response syndrome (SIRS), admission to a medical, surgical or cardiac ICU, and not receiving any form of renal replacement therapy. Patients were enrolled within 24 hours of ICU admission. Exclusion criteria included inability to provide informed consent, non-English speaking, metastatic cancer, severe immunosuppression, vulnerable populations, and do not resuscitate or comfort care orders within the first 24 hours of ICU admission. For the purposes of this ancillary study, we selected the first 170

		CITRC participants who had available spot urine and blood samples within 24 hours of ICU admission. All urine samples were collected in patients with an indwelling urinary catheter.<br><br>**The Healthy Kidney Study (HKS).** For comparison, we obtained blood and spot urine samples from 70 participants in the Healthy Kidney Study, a study of individuals who were free of clinically apparent kidney disease, defined by an estimated glomerular filtration rate (GFR) ≥90 ml/min/1.73m2 and a urine albumin to creatinine ratio <30 mg/g. The HKS recruited persons from general and family medicine clinics throughout the UW system between 2012 – 2016.
Setting	5	Describe the setting, locations, and relevant dates, including periods of recruitment, exposure, follow-up, and data collection<br>Measurement of Outcomes<br><br>CITRC research coordinators identified the initiation of inpatient dialysis and death by chart review, inpatient visits and telephone callbacks to patients. Outcomes were adjudicated until 28 days after ICU admission. The primary outcome MAKE28 was defined as either (1) doubling of serum creatinine concentration from the creatinine measurement at CITRC study enrollment, (2) receipt of any renal replacement therapy, or (3) death. We selected MAKE28 to consider the breadth of clinical consequences in the ICU and to account for issues of competing risk due to death (30).<br><br>Measurement of Other Study Data<br>AKI at study enrollment was defined as a ≥ 0.3 mg/dL and/or ≥ 50% increase in SCr measurement at the time of study enrollment compared to the baseline SCr value measured prior to study enrollment or a decrease in urine output(31). We determined the baseline creatinine value using a hierarchical approach in which creatinine values obtained during the year prior to the index hospitalization were given priority over in-hospital measurements obtained before study enrollment. We measured creatinine in blood and urine samples and cystatin C in blood samples collected from participants at study enrollment in CITRC. Serum and urine creatinine concentrations were measured using the Modified Jaffe Method and plasma cystatin C concentrations were measured using an immunoturbidimetric assay (Gentian AS, Moss Norway) on a Beckman DXC Unicell clinical analyzer. Prevalent diabetes was defined by participant self-report of diabetes or the use of insulin. CKD stage III or greater as a glomerular filtration rate less than 60 ml/min per 1.73 m2 as calculated by the Chronic Kidney Disease Epidemiology (CKD-EPI) Collaboration equation(32) using a patient's baseline creatinine prior to hospitalization or based on chart diagnosis. We defined septic shock as meeting Sepsis-3 criteria and hypotension(33). Mechanical ventilation and vasopressors were defined anytime during ICU admission requiring these therapies.
Participants	6	(*a*) Give the eligibility criteria, and the sources and methods of selection of participants. Describe methods of follow-up<br>Eligibility included meeting at least two of four criteria for the systemic inflammatory response syndrome (SIRS), admission to a medical, surgical or cardiac ICU, and not receiving any form of renal replacement therapy. Patients were enrolled within 24 hours of ICU admission. Exclusion criteria included inability to provide informed consent, non-English speaking, metastatic cancer, severe immunosuppression, vulnerable populations, and do not resuscitate or comfort care orders within the first 24 hours of ICU admission.
		(*b*) For matched studies, give matching criteria and number of exposed and unexposed<br>*N/A*
Variables	7	Clearly define all outcomes, exposures, predictors, potential confounders, and effect modifiers. Give diagnostic criteria, if applicable<br>We originally selected 15 candidate secretory solutes based on extensive literature review and discussion with colleagues in this field. Literature review criteria included at least one of the following : specificity for organic anion transporters (OAT1, OAT3), a high degree of protein-binding, suggesting minimal glomerular filtration, and/or kidney clearances that substantially exceed that of creatinine, a primarily filtered substance, and potential for accurate measurement by our LC/MS methods.10-12<br><br>We selected adjustment variables *a priori* on the basis of biologic plausibility and

		prior literature suggesting these variables may confound associations between U/P ratios of secretory solutes and clinical outcomes. The first adjusted model included age, sex, Black race, urine albumin to creatinine concentrations (to account for differences in urine volume), AKI status on study enrollment, and sequential organ failure score (SOFA) excluding the renal component(34). The SOFA score was based on maximal variables obtained during the first day of ICU admission. The second model added adjustment for SCr or cystatin c concentrations measured at CITRC study enrollment. Because we tested eight different associations between secretory solutes (seven secretory solutes and the composite secretion score) and kidney events, we chose the conservative estimate of a Bonferroni-corrected *p value* cutoff of 0.05/8 = 0.00625 to declare significance. We also performed subgroup analyses to explore whether the size of associations between the composite secretion score and MAKE28 differed by AKI status on study enrollment, shock, and sepsis-3. All analyses were performed using Stata release 15.1 software (StataCorp, College Station, TX, USA).
Data sources/ measurement	8*	For each variable of interest, give sources of data and details of methods of assessment (measurement). Describe comparability of assessment methods if there is more than one group<br>We measured total plasma concentrations of secretory solutes using protein precipitation, solid phase extraction, and LC-MS/MS and spot urine concentrations using solid phase extraction and LC-MS/MS. Calibration was achieved using a single point calibration approach to correct for potential drift that may be caused by changes in reagents, calibrator lots, equipment, or settings. The peak areas of each solute were normalized to peak areas of labeled internal standards added to each well. Peak area ratios were then standardized to single point calibrators (mean of five replicates run performed on each plate). We have previously determined accurate concentrations of each solute in the single point calibrators (pooled human serum and urine) by standard addition of solutions of pure compounds analyzed by quantitative nuclear magnetic resonance. Laboratory coefficients of variation were generally low (**Supplemental Table S1**). We have found no changes in the concentrations of these solutes with up to three freeze-thaw cycles.<br><br>Urinary Biomarker Measurements<br>We measured urine samples for urinary kidney injury molecule 1 (KIM-1) and neutrophil gelatinase associated lipocalin (NGAL) using a multiplex assay (Meso Scale Discovery (MSD)). Biomarkers were measured in one batch and the intraplate coefficient of variation for KIM-1 was 15% and for NGAL was 13%.
Bias	9	Describe any efforts to address potential sources of bias<br>We performed empiric protein binding studies in our laboratory from samples of patients with critical illness, which revealed lower protein binding percentages than previously reported for three solutes (isovalerylglycine, tiglylglycine, and xanthosine) among critically ill adults
Study size	10	Explain how the study size was arrived at<br>Based on our prior literature that a minimum of 150 patients would show a difference in urinary biomarker concentrations for AKI in critical illness.
Quantitative variables	11	Explain how quantitative variables were handled in the analyses. If applicable, describe which groupings were chosen and why<br>To facilitate presentation of participant characteristics and provide a single metric for summarizing associations, we computed a composite secretion score as the average of the individual U/P ratios. To account for right skewed data, we log-transformed the individual solute clearances. To ease comparison of different solutes, we standardized or rescaled solute measurements to have a mean of 0 and a standard deviation of 1. We then computed the composite secretion score as the average of the seven U/P ratios.
Statistical methods	12	(*a*) Describe all statistical methods, including those used to control for confounding<br>We selected adjustment variables *a priori* on the basis of biologic plausibility and prior literature suggesting these variables may confound associations between U/P ratios of secretory solutes and clinical outcomes. The first adjusted model included age, sex, Black race, urine albumin to creatinine concentrations (to account for differences in urine volume), AKI status on study enrollment, and sequential organ failure score (SOFA) excluding the renal component(34). The SOFA score was based on maximal variables obtained during the first day of ICU admission. The second model added adjustment for SCr or cystatin c concentrations measured at CITRC study enrollment. Because we tested eight different associations between secretory

		solutes (seven secretory solutes and the composite secretion score) and kidney events, we chose the conservative estimate of a Bonferroni-corrected *p value* cutoff of 0.05/8 = 0.00625 to declare significance.
		(*b*) Describe any methods used to examine subgroups and interactions<br>We also performed subgroup analyses to explore whether the size of associations between the composite secretion score and MAKE28 differed by AKI status on study enrollment, shock, and sepsis-3.
		(*c*) Explain how missing data were addressed<br>No missing data for key exposures, confounders or outcomes.
		(*d*) If applicable, explain how loss to follow-up was addressed<br>N/A
		(*e*) Describe any sensitivity analyses<br>In sensitivity analyses, we stratified patients with AKI on study enrollment into etiologies of AKI (pre-renal and acute tubular necrosis (ATN)) based on the fractional excretion of filtered sodium (FENa). Among 45 patients with AKI on study enrollment, 18 had pre-renal AKI and 15 had ATN. The U/P ratio of tubular solutes were 2-3 fold higher in patients with pre-renal AKI compared to ATN (**Table S8**). We also compared U/P ratios and fractional excretions of tubular solutes. We found that both a higher U/P ratio and a higher fractional excretion of tubular solutes were associated with a lower risk of MAKE28. These findings provide reassurance that there was net secretion of each of these solutes (**Table S9**).
**Results**		
Participants	13*	(a) Report numbers of individuals at each stage of study—eg numbers potentially eligible, examined for eligibility, confirmed eligible, included in the study, completing follow-up, and analysed<br>170 participants were eligible in CITRC with matched blood and urine samples. We used all 170 participants.
		(b) Give reasons for non-participation at each stage<br>n/a
		(c) Consider use of a flow diagram<br>n/a
Descriptive data	14*	(a) Give characteristics of study participants (eg demographic, clinical, social) and information on exposures and potential confounders<br>Among participants in the CITRC cohort, the mean age was 50 years, 67% were men, and 79% self-identified as White race (**Table 1**). CITRC participants had numerous comorbidities, including diabetes (33%), hypertension (38%) and CKD (7%). Diagnosis of sepsis-3 was common (85%) as was the use of mechanical ventilation (49%) and septic shock (49%). The mean SOFA score on day 1 was 7.1 ± 4.5 and the mean SCr concentration on day 1 was 1.3 ± 1.0 mg/dL. Among participants in the HKS cohort, the mean age was 50 years; 50% were male and the mean SCr concentration was 0.82 ± 0.19 mg/dL.
		(b) Indicate number of participants with missing data for each variable of interest<br>Mentioned above
		(c) Summarise follow-up time (eg, average and total amount)<br>28 day outcomes
Outcome data	15*	Report numbers of outcome events or summary measures over time<br>The primary outcome of MAKE28 occurred in 50 (29%) participants, with a qualifying event of death in 11, requirement for dialysis in 5, and a doubling in serum creatinine in 34.
Main results	16	(*a*) Give unadjusted estimates and, if applicable, confounder-adjusted estimates and their precision (eg, 95% confidence interval). Make clear which confounders were adjusted for and why they were included<br>In fully adjusted models, each standard deviation higher composite secretion score was associated with a 25% lower risk of MAKE28 (relative risk, 0.75; 95% CI 0.62 to 0.91; $p<0.001$). Individually, higher U/P ratios of isovalerylglycine and tiglylglycine were associated with the development of MAKE28 even after correction for multiple testing (RR=0.72; 95% CI 0.57 to 0.91 and RR=0.70; 95% CI 0.58 to 0.84, respectively). In sensitivity analyses, associations were not materially altered by adjusting for cystatin C instead of SCr or by adjusting for the ratio of urine to serum creatinine **(Supplement Tables S4 and S5).** The composite secretion score was also associated with doubling of serum creatinine or dialysis within 7 days of study enrollment **(Supplement Table S6).** Plasma concentrations of tubular solutes were associated with the development of MAKE28, suggesting renal retention is reflected by elevated plasma concentrations (**Supplement Table S7**).

		(*b*) Report category boundaries when continuous variables were categorized
		(*c*) If relevant, consider translating estimates of relative risk into absolute risk for a meaningful time period
Other analyses	17	Report other analyses done—eg analyses of subgroups and interactions, and sensitivity analyses<br>In exploratory subgroup analyses, the size of associations between the composite secretion score and MAKE28 were statistically similar across strata defined by sepsis-3 status and septic shock on study enrollment. In contrast, the risk of MAKE28 was statistically different between patients with and without AKI on study enrollment (*p value for interaction 0.03*). Patients with AKI on study enrollment had a reduced risk for MAKE28 with higher composite secretion score but in patients without AKI there was no significant difference in MAKE28 outcomes based on the composite secretion score. (**Table 4**).
**Discussion**		
Key results	18	Summarise key results with reference to study objectives<br>We found lower urine to plasma ratios of endogenous secretory solutes in critically ill patients compared with healthy control subjects. Among critically ill patients, higher urine to plasma ratios of isovalerylglycine and tiglylglycine, and a higher composite secretory score, were associated with a lower risk of MAKE28 after controlling for demographics, ICU severity of illness, SCr and urinary biomarkers (KIM-1 and NGAL). Our findings suggest that measurement of proximal tubular secretion may provide additional diagnostic and prognostic information about kidney function in critical illness that complements measures of glomerular filtration (e.g. creatinine). Assessment of both secretion and filtration in the ICU could improve risk stratification of patient populations and improve phenotyping of AKI in the ICU. To our knowledge, this is the first study in critically ill patients to characterize tubular secretory function and determine the independent association with clinical outcomes.
Limitations	19	Discuss limitations of the study, taking into account sources of potential bias or imprecision. Discuss both direction and magnitude of any potential bias<br>This study includes several important limitations. First, we used spot urine to plasma ratios of secretory solutes as a surrogate for timed clearances. However, timed urinary clearances, as performed in stable outpatients, require steady-state plasma concentrations and are less accurate when kidney dysfunction is evolving(28). Moreover, in outpatients we have shown that supervised 10-hour timed urine collections and spot urine to plasma measurements in the same people on the same day corresponded very closely(21). Second, laboratory measurement error in determining solute concentrations in blood and urine may have falsely exaggerated the lack of correlation between tubular secretion relative to creatinine. However, laboratory precision was generally high and such misclassification would be expected to be non-differential and bias associations to the null. In contrast, we found that tubular secretion was strongly associated with the development of MAKE28. Third, we did not perform goal standard measurements of filtration using exogeneous markers, such as iothalamate, but instead relied on clinically used markers, such as creatinine, and to a lesser extent, cystatin C. Fourth, the small sample size and single study center hinders generalizability of the results. However, it was reassuring that the point estimate for each secretory solute consistently demonstrated a decreased risk for MAKE with an increase in secretory solute U/P ratios. Future work in larger cohorts may allow distinguishing differences in risk of clinical outcomes between individual tubular secretory solutes.
Interpretation	20	Give a cautious overall interpretation of results considering objectives, limitations, multiplicity of analyses, results from similar studies, and other relevant evidence<br>In conclusion, this study demonstrates the first characterization of proximal tubular secretion as an independent marker of kidney function in a longitudinal cohort of critically ill participants. These data motivate additional investigation of tubular secretion as a measure of kidney function, with potential applications in early identification of AKI complications and outcomes in critical illness and improving dosing of ICU drugs that undergo proximal tubular secretion.
Generalisability	21	Discuss the generalisability (external validity) of the study results<br>Fourth, the small sample size and single study center hinders generalizability of the results. However, it was reassuring that the point estimate for each secretory solute consistently demonstrated a decreased risk for MAKE with an increase in secretory solute U/P ratios. Future work in larger cohorts may allow distinguishing differences in risk of clinical outcomes between individual tubular secretory solutes.

**Other information**		
Funding	22	Give the source of funding and the role of the funders for the present study and, if applicable, for the original study on which the present article is based<br><br>**Funding Support:** PKB was supported by grants from the Digestive and Kidney Diseases K23DK116967, the University of Washington Diabetes Research Center P30DK017047, and an unrestricted gift to the Kidney Research Institute from the Northwest Kidney Centers. EDS was supported by the Vanderbilt O'Brien Kidney Center (NIDDK 5P30 DK114809-03) The funding sources had no role in design and conduct of the study; collection, management, analysis, and interpretation of the data; and preparation, review, or approval of the manuscript.

*Give information separately for exposed and unexposed groups.

Note: An Explanation and Elaboration article discusses each checklist item and gives methodological background and published examples of transparent reporting. The STROBE checklist is best used in conjunction with this article (freely available on the Web sites of PLoS Medicine at http://www.plosmedicine.org/, Annals of Internal Medicine at http://www.annals.org/, and Epidemiology at http://www.epidem.com/). Information on the STROBE Initiative is available at http://www.strobe-statement.org.
